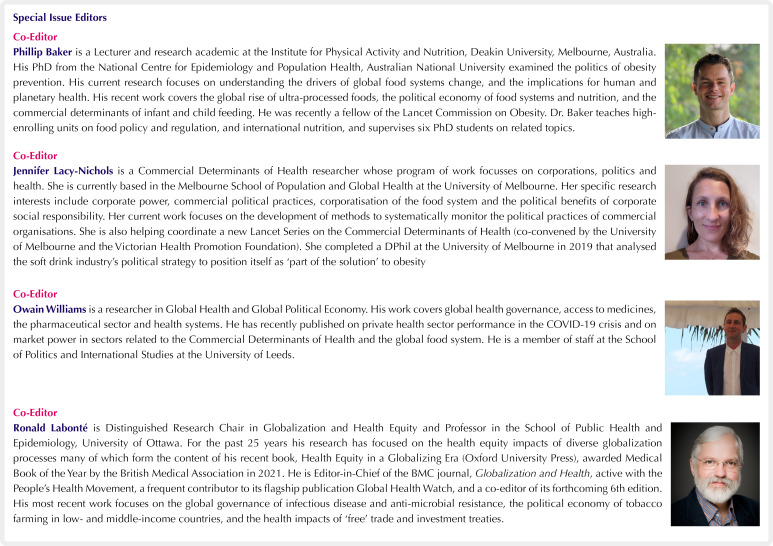# The Political Economy of Healthy and Sustainable Food Systems: An Introduction to a Special Issue

**DOI:** 10.34172/ijhpm.2021.156

**Published:** 2021-11-14

**Authors:** Phillip Baker, Jennifer Lacy-Nichols, Owain Williams, Ronald Labonté

**Affiliations:** ^1^Institute for Physical Activity & Nutrition, Deakin University, Geelong, VIC, Australia.; ^2^Melbourne School of Population and Global Health, University of Melbourne, Melbourne, VIC, Australia.; ^3^School of Politics and International Studies, University of Leeds, Leeds, UK.; ^4^School of Public Health and Epidemiology, University of Ottawa, Ottawa, ON, Canada.

**Keywords:** Politics, Power, Political Economy, Food Systems, Nutrition, Commercial Determinants

## Abstract

Today’s food systems are contributing to multiple intersecting health and ecological crises. Many are now calling for transformative, or even radical, food systems change. Our starting assumption in this Special Issue is the broad claim that the transformative changes being called for in a global food system in crisis cannot – and ultimately will not – be achieved without intense scrutiny of and changes in the underlying political economies that drive today’s food systems. The aim is to draw from diverse disciplinary perspectives to critically evaluate the political economy of food systems, understand key challenges, and inform new thinking and action. We received 19 contributions covering a diversity of country contexts and perspectives, and revealing inter-connected challenges and opportunities for realising the transformation agenda. We find that a number of important changes in food governance and power relations have occurred in recent decades, with a displacement of power in four directions. First, *upwards* as globalization has given rise to more complex and globally integrated food systems governed increasingly by transnational food corporations (TFCs) and international financial actors. Second, *downwards* as urbanization and decentralization of authority in many countries gives cities and sub-national actors more prominence in food governance. Third, *outwards* with a greater role for corporate and civil society actors facilitated by an expansion of food industry power, and increasing preferences for market-orientated and multi-stakeholder forms of governance. Finally, power has also shifted *inwards* as markets have become increasingly concentrated through corporate strategies to gain market power within and across food supply chain segments. The transformation of food systems will ultimately require greater scrutiny of these challenges. Technical ‘problem-solving’ and overly-circumscribed policy approaches that depoliticise food systems challenges, are insufficient to generate the change we need, within the narrow time-frame we have. While there will be many paths to transformation, rights-based and commoning approaches hold great promise, based on principles of participation, accountability and non-discrimination, alongside coalition building and social mobilization, including social movements grounded in food sovereignty and agroecology.

## Introduction

The food systems we inherit today are incredible human achievements. Over the past century, substantial increases in world food production contributed to steady declines in undernutrition and food insecurity for billions. Yet we have never been more aware of the threats that food systems now pose for human and planetary health. Against a backdrop of rapid economic and social transition, many countries are now experiencing rapid dietary change and multiple burdens of undernutrition, obesity and non-communicable diseases.^1^ Over a decade since the 2008-2009 global food price crisis, insufficient and precarious access to nutritious food remains an ever-present danger for millions, a situation now greatly exacerbated by the unfolding coronavirus disease 2019 (COVID-19) crisis. Since 2014, world hunger (undernourishment) has been rising, after decades of steady decline, affecting one in 10 people (768 million in total) in 2020, mainly through the impacts of climate change, conflict and economic insecurity.^2^ Food systems are also a leading driver of global environmental including through deforestation, biodiversity loss and water pollution, while generating up to 30% of greenhouse gas emissions.^3^ As the world’s population grows to an estimated 10 billion people by 2050, with more people becoming richer and more urbanised, these challenges will become more pressing.^3^

Recognising these challenges, the idea of ‘sustainable diets’ and ‘sustainable food systems’ – those that deliver “food security and nutrition for all in such a way that the economic, social and environmental bases to generate food security and nutrition for future generations are not compromised” – has jumped into scientific, social and policy lexicons.^4^ Many are now calling for transformative and urgent, some even say radical, food systems change. The High Level Panel of Experts (HLPE) of the Committee on World Food Security (CFS) calls for a new global narrative that prioritizes the right to food, and critical policy shifts, including food production practices and governance grounded in agroecology.^5^ The landmark EAT-Lancet Commission on Food, Planet and Health calls for a ‘Great Food Transformation’ as essential to keeping global society within health and earth-systems boundaries.^3^ Analyses by the Lancet Commission on Obesity, show how industrial food systems are driving a Global Syndemic of undernutrition, obesity and climate change, and calls for new forms of governance and business operating models, that address food systems power asymmetries.^6^

Yet the characteristic features of food systems problems pose many challenges for this ‘transformation agenda.’ Diffuse causes can render them invisible to affected communities and decision-makers, made more problematic in the context of globalization and the distancing of regulators and citizens from localities experiencing the environmental and social harms of food production. The ‘slow-burning’ accumulation of environmental (eg, climate change) and nutritional risks (ie, across the life-course and inter-generationally) are mismatched with short-term political cycles and shareholder returns geared for quick and demonstrable wins. With the exception of hunger and its relationship with political destabilisation, food issues tend to have low-level visibility with political leaders relative to more tangible vote-winning ones (eg, education, the economy and infrastructure). Most importantly food systems harms disproportionately burden politically marginalised and diffuse groups – namely children, women and the poor^7,8^ – in contrast to the concentrated and orchestrated power of governments and food industry groups that often contribute to and even profit from those harms.

Our starting assumption in this Special Issue is the broad claim that the transformative changes being called for in a global food system in crisis cannot – and ultimately will not – be achieved without intense scrutiny of and changes in the underlying political economies that drive today’s food systems. Multiple reports, as well as the recent United Nation Food Systems Summit (UNFSS), propose hundreds of potential actions for achieving healthy and sustainable food systems. However, with some key exceptions,^9^ there has been much less attention to the deeper question of how we achieve the transformative changes being called for, nor to the question of who or what might enable or impede those changes going forward. This is a crucial omission given the potential scope and political complexity of any food systems transformation agenda. These observations strongly justify the use of an integrated political economy and food systems approach that considers actors, interests, structures and power as key explanatory variables for understanding today’s food challenges and transformative potential. By political economy we mean the interplay between political, economic and social forces in society, the distribution of power and resources between different individuals and groups within and surrounding food systems, and the structures and processes that generate, sustain or transform these relationships over time.^10^

 We proceed from a basic premise that the political economy of food systems is under-theorized, with robust empirical and strong disciplinary approaches so far lacking comparative or overarching focus in existing literature. Furthermore, that overly-technocratic and compartmentalised ‘problem solving’ approaches that ignore the role of political economy are inadequate to address the scale of food systems challenges we face, within the increasingly urgent time-frames we have. New approaches to scholarship are needed, including more reflexive ideas of what constitutes genuinely critical and transformative knowledge production. This Special Issue seeks to make a contribution that addresses these gaps and theoretical deficits. The aim is to draw from diverse disciplinary perspectives to critically evaluate the political economy of today’s food systems, understand key challenges to the transformation agenda, and inform new thinking and action towards realising meaningful food system change. Several key questions are addressed: What does it mean to transform a food system from a governance, policy and regulatory standpoint? Who stands to win and who to lose from such a transformation? What might enable or impede this transformation in the context of existing political and economic systems? What might a transformative political economy of food systems ultimately look like, and how might we achieve it?

## Approach

This Special Issue builds on a workshop involving many of the contributors held at the University of Sydney in July 2019, as a side event to the Food Governance Conference, hosted by the Sydney Law School. Understanding the complexity of food systems political economy, necessitates a multi-disciplinary approach.^11^ Acknowledging this, we draw upon and weave together contributions and themes from scholars across disciplines, including food policy and governance, public health, international political economy, political science, sociology, law and regulatory studies. Many of these contributions draw from novel empirical sources and use cutting-edge methodologies. Finally, today’s food systems challenges are global in scope. In putting together this collection, we endeavoured to capture and select studies and authors from a range of countries and regions across the Global North and South.

Food systems are complex adaptive systems, comprising many inter-connected, multi-layered and dynamic elements, characterised in terms of inter-dependencies, non-linear feedback loops and emergent properties.^12,13^ Through this lens, the ‘global food system’ is in reality a ‘system of systems’ spanning global, national and sub-national levels with significant variations between and within countries, and with strong multi-level interconnectedness. Several ‘typologies’ distinguish between food system types at the country level ranging from industrial systems in highly urbanized countries (eg, Australia, the United States), through to mixed (eg, Germany, Italy), transitioning (eg, China, Brazil) and rural or agrarian (eg, Indonesia, Kenya).^4,14^ Some use the term ‘alternative food system’ as distinct from, or in resistance to, a dominant (typically capitalist-industrial) system, or urban food systems in contrast to rural or peri-urban ones, or to the Indigenous food systems of First Nations peoples. There is, therefore, no singular food systems political economy but many, with diverse configurations of actors, interests and relations of power.

Through a systems lens, power can be conceptualised as exercised overtly and directly between actors within food systems, but also indirectly in more hidden or invisible ways, and as an emergent and dynamic property of the system itself.^15^ As International Panel of Experts on Sustainable Food Systems (IPES-Food) describe it, a systems approach to power ‘…reinforces the need to train our attention on food systems as a whole, and on the broader political and economic systems in which they are embedded, in order to capture the webs of self-reinforcing power and influence that create systemic dynamics and systemic lock-ins.’^11^

The papers in this Special Issue draw upon different conceptualisations of power, and explore diverse food systems topics and issues. To help integrate these concepts and organize the contributions thematically, we draw from the review of power by Walls, Harris and Nisbett in this Special Issue,^16^ and build on their adaptation of Gaventa’s Power Cube model.^15,17^ Shown in Figure, this three-dimensional model is particularly useful for understanding intersecting dimensions of power as it relates to complex, multi-level food systems actor networks.^10^ It depicts levels (global, national, sub-national), spaces (closed, invited, claimed), and forms (instrumental, structural, discursive, and material) of power, with each side of the cube representing a continuum rather than a static set of possible categories.^15^

**Figure:**
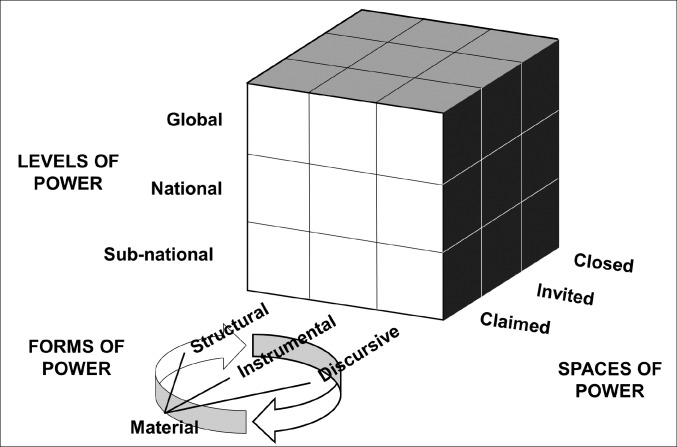
The Power Cube Model Showing Levels, Spaces and Forms of Power. Notes: Adapted with permission from Gaventa^15^; we adopted instrumental, structural and discursive concepts of power, given their comprehensive theoretical development and application in the food and political science literatures^18,19^; which differs to the visible, hidden and invisible forms of power in the original Powercube model.

## Understanding Power Within Food Systems and Challenges to Food Systems Transformation

 In total we received 19 submissions. These contributions fall under several broad approaches and themes, and they cover a diversity of country contexts and perspectives, revealing inter-connected challenges and opportunities for realising the food systems transformation agenda. In the following sections, we combine a summary of existing literature with these contributions, to understand changes in the different dimensions of food systems power identified in the Power Cube model, and the implications of these changes for the transformation agenda.

###  Levels of Power: A Shift Upwards and Downwards Away From the National

The first dimension of the model is consistent with a ‘multi-level’ conceptualisation of food systems governance whereby power can manifest at a given level (ie, horizontally) and/or across multiple levels (ie, vertically) along a continuum from the local to the global.^13,20^ In recent decades the loci of food systems power has undoubtedly shifted *upwards* from the national to the global level, underpinned by processes of economic globalization. Trade liberalization has resulted in the systematic reduction in barriers to cross-border food trade and investment, bound governments to expanding international trade laws, and limited the scope of regulatory measures they have available to achieve domestic policy objectives. The inclusion of agriculture in the General Agreement on Tariffs and Trade in 1994, the establishment of the World Trade Organization (WTO) in 1995, and since then an explosion of regional and bilateral trade agreements has widened the scope of international trade rules to encompass trade in food commodities and food-related services, with enforceable rules governing investment and intellectual property rights, all of which discipline government measures that could restrict trade.^18,21,22^ In parallel, many countries have liberalised their economies unilaterally alongside the spread of neoliberal ‘free-market’ thinking and economic policies emphasising market liberalization, privatization of state owned enterprises, and market- over state-led approaches to governance. The same thinking has underpinned, and indeed been actively promoted by, the structural adjustment programmes of the World Bank and International Monetary Fund.^23,24^

These globalization processes have helped to facilitate an unprecedented increase in the number, size and reach of transnational food corporations (TFCs) and in turn the expansion and integration of their cross-border food production and supply chain networks.^18,23,25,26^ In this issue, for example, Moodie et al, in their analysis of the transnational ultra-processed food industry, show that as markets have stagnated in their high-income country home markets, TFCs have vigorously pursued expansion into countries of the Global South, attracted by their high economic growth rates, and young and increasingly affluent populations with urbanizing lifestyles.^27^ With this has come the expansion of global food marketing and advertising activities as TFCs enter into new markets throughout the Global South, powerfully shaping beliefs about what foods are normal and socially desirable.^28^

Financialization is another central feature of the restructuring of the global food economy, involving the emergence of a liberal financial regime, characterised by rapid growth in marketized securities and monetary exchange freedoms.^29,30^ A small number of private equity firms located in the Global North (eg, Blackrock, Vanguard and Fidelity) have funnelled vast amounts of equity into publicly listed food corporations, providing them with finance for expansion and market consolidation.^31^ Smith and Lawrence show, in their case study on the Australian sugar industry, how expanding food commodity futures trading and speculative farmland investment by financial actors (eg, private equity, pension and sovereign wealth funds) is driving the concentration of power within agro-industrial food supply chains.^32^

The implications of economic globalization for sustainable food systems are contested.^33^ Proponents claim, with some caveats, this enhances food security and economic development by offering low- and middle-income country producers access to larger markets and foreign investment, and hence improved economic development, income growth and state tax revenues. It leads to overall systems efficiency by promoting competition and regional/country specialisation in the production of food commodities based on natural resource endowments. Food surpluses in more endowed producer countries can address food deficits in those where population growth has outstripped quantitative gains in food production, and expand year-round access to nutritious foods. Opponents say that while trade is needed, and even desirable, the regime infringes on the sovereignty of nations and peoples to determine their own food policies, conflicts with the right to healthy and culturally-appropriate food, and devalues food security, livelihoods and other non-market dimensions of food systems.^34^ Increasing distances between localities of production and points of consumption has reduced the visibility of the social and environmental externalities of production processes, and economic relationships in globalized supply chains.^29,35^ The growth of TFCs makes free trade efficiencies irrelevant as they capitalise on the comparative advantages of different production localities within their globalized supply chains, and increasingly dictate prices as they command greater market power. Furthermore, a ‘double-standard’ of protectionism exists, given the large producer subsidies under the US Farm Bill and European Union **(**EU) Common Agricultural Policy, while LMICs are required to open their markets to highly capitalized foreign competition.^24^

Several contributors to this Special Issue show how the evolving trade and investment regime influences the ‘regulatory space’ governments have available to achieve their domestic food systems objectives. Garton et al show how Free Trade Agreement rules have impeded regulatory interventions targeting food environments, although with variable outcomes, ranging from policy being modified (eg, Thailand’s nutrition warning labels), delayed (eg, Indonesia’s nutrition warning labels), or abandoned (eg, Samoa’s turkey tail ban).^36^ Importantly, they report instances where policy was upheld (eg, nutrition labelling in Mexico), and urge governments not to be deterred from implementing evidence-informed policies. Russ et al show how interventions in the WTO have constrained worldwide implementation of the World Health Organization’s (WHO’s) International Code of Marketing of Breastmilk Substitutes (BMS). Between 1995 and 2019, large dairy-producing and exporting member states (mostly the US, EU, Australia, New Zealand), made 245 interventions against the BMS regulations of other member states, largely through interventions in WTO Committees and other sub-arbitration processes. Many cited deviations from standards established by the Codex Alimentarius Commission (CAC), the United Nation (UN) food standard-setting body, where the same member states and industry groups also intervened to narrow the scope of relevant CAC standards. While these cases show how actors within the global trade regime can directly reduce the regulatory space of national governments, there is also likely to be a much wider ‘chilling’ effect on others, as the implied threat of trade sanctions or costly arbitration can deter commitment to action.^37^

As power within food systems has shifted upwards to the global, the governing role of inter-governmental organizations is prominent, if not more important than ever. Multilateral organizations play key roles, by convening actors and establishing international agreements, norms and accountability frameworks to govern the global food system in the absence of binding international law. These include the specialised food and nutrition agencies of the UN System – the Food and Agricultural Organization (FAO), WHO, United Nations International Children’s Emergency Fund (UNICEF), World Food Programme and the International Fund for Agricultural Development, as well as various human rights bodies. The CFS, underwent significant reform following the 2008/2009 world food price crisis to become the world’s foremost multi-lateral and participatory food governance platform, including mechanisms for convening civil society and market actors, and for scientific input through the HLPE. Yet, these organizations have been subject to increasing pressure to engage with non-state actors, especially the private sector. This has, at times, been highly visible and politicised, for example in the contested negotiations over WHO’s Framework for Engagement with Non-state Actors. Furthermore, although in principle these multi-lateral organizations answer to all UN member states, major Western donors with large agri-food industries have disproportionate power in agenda-setting and decision-making. For example, over decades, the US government has challenged the development of WHO policies and technical standards on sugar, unhealthy diets, the regulation of marketing breastmilk substitutes, and managing conflicts of interest in nutrition policy, among others.^38,39^

As power has shifted upwards away from the national, it has simultaneously shifted *downwards* to the sub-national. Food governance scholars describe this as the ‘re-scaling’ of food policy to the local level whereby in response to rapid urbanization and the decentralisation of authority underway in many countries, sub-national actors and in particular municipal governments are taking on more prominent roles in food governance.^40^ Increasing recognition of the important role of cities has led to the establishment of international city networks to foster commitment, cooperation and learning (eg, the Milan Food Policy Pact involving ~140 signatory cities, and the C40 network of cities concerned with climate change), as well as national networks (eg, the UK’s Sustainable Food Cities).^41^ Many cities have established food policy councils as structures for multi-stakeholder engagement and developed urban food policies targeting single or multiple sustainability issues.^41,42^ However, the scale and pace of urbanization in many countries throughout the Global South creates substantial governance challenges. Although many countries of the Global North have had majority urban populations since the 1950s, most in Africa and Asia are still predominantly rural with large informal food economies. Importantly, political processes and governance institutions take time to evolve, and the policy and regulatory frameworks needed to regulate increasingly complex urban food systems may not keep pace with regulatory demand in these contexts.^43^

###  Spaces of Power: Closed, Invited and Claimed 

As power has shifted both upwards and downwards, it has also shifted *outwards,* as non-state actors have come to play increasingly important roles in food governance. Here we can engage with the Power Cube concept of spaces, the institutional channels and policy-making arenas in which food system actors interact, make decisions and take action.^16^ Power determines who participates and who does not in decision-making, and defines the boundaries of potential action.^15^ Invited spaces are those that are inclusive, where stakeholders can participate to inform decision-making although agendas may be pre-determined by the powerful. Closed spaces are those of exclusion, where the powerful make decisions behind closed doors making it difficult or impossible for others to have influence. Spaces are claimed when actors capture existing or create newly autonomous arenas for engagement and action. Spaces may differ in membership, issue focus, the rules governing conduct, and so on. They may be institutionalised and ongoing (eg, formal governance bodies) or more transient and temporary (eg, consultations, technical meetings), or represent spaces of material exchange in food supply chains.

The concept of invited spaces aligns closely with ‘participatory’ and ‘reflexive’ forms of governance viewed by many as crucial to fostering dialogue, learning and accountability among food actors, and to formulating integrated and adaptable policies that address the dynamic complexity of food systems challenges.^44-46^ A diversity of state-anchored institutional designs have emerged as invited spaces for sustainable food governance at global, national and sub-national levels. As mentioned earlier, the CFS is the world’s foremost inclusive intergovernmental and multi-stakeholder platform, while at national and (mostly) sub-national levels food policy councils have emerged since the early 1980s for convening food systems actors. These address a wide range of issues from the singular (eg, food security, nutrition) to more integrated and multi-issue approaches.^41,47,48^ At the national level, some countries have developed inclusive governance models. For example, Brazil’s National System of Food Security and Nutrition was a multi-sector and multi-level institutional framework that underpinned its success in driving down malnutrition rates, enabled by strong social policy reform and economic growth.^49,50^

The recent UNFSS illustrates how governance spaces can be captured or claimed by corporate interests. Whereas previous UN food summits were largely multi-lateral and nation-state led, the UNFSS was initiated through a partnership between the UN Secretariat and the World Economic Forum, an organization that convenes the world’s largest corporations, and promotes multi-stakeholder capitalism as an approach to addressing global challenges. Although branded a ‘people’s summit,’ the UNFSS gave corporations and their trade associations a privileged role in the process, by engaging them across various settings and pre-Summit events.^51^ In many (mostly high-income) countries and globally, public-private partnerships or multi-stakeholder partnerships (MSPs) have emerged as new ‘middle-spaces’ of governance, reflecting rising preferences for more market-oriented and private-sector engaged approaches ^52,53^. For example, established in 2010, the Scaling Up Nutrition movement is a MSP involving UN, donor, civil society and business networks, and now operates across 60 countries, and three Indian States.^54^ Another is the UN Global Compact, launched in 2000 as the world’s largest corporate social responsibility initiative involving >8000 corporate entities across 170 countries including many of the world’s largest food companies.^55^

Fanzo et al, in this Special Issue, conduct an analysis of public-private partnerships in nutrition. They conclude that state and civil society actors engaging with the private-sector must ensure that potential, perceived or actual commercial conflicts of interest are addressed. They also find that substantial trust deficits exist between public and private actors in food systems, and that there is no compelling evidence to justify using partnerships over legislative policy approaches to achieve desired outcomes.^56^ Others find, that the participation of corporate actors in policy agenda-setting and decision-making can ‘depoliticise’ food problems by enrolling others in negotiations, resulting in compromise and weaker outcomes (eg, voluntary rather than mandatory regulation), and solutions that preference corporate interests (eg, reformulation, but not marketing restrictions).^52,57^ The recent UNFSS, for example, was described as ‘strategically silent’ on the problem of market concentration and corporate power within food systems, mentioning this issue only infrequently, and advanced innovation and technology-based solutions over transformative structural change.^58^ At the national-level, the Australian government established the Healthy Food Partnership, an MSP with a highly-circumscribed focus on the reformulation and labelling of packaged foods.^59^

At the same time, businesses have increasingly claimed new spaces of governance by establishing private consortia and multi-stakeholder alliances to set private sustainability standards, in many cases in unison with civil society groups and completely outside of government involvement (eg, the Roundtable on Sustainable Palm Oil).^60^ These more devolved forms of regulation largely seek to make existing actor arrangements and institutions work better, rather than challenge systems structures and transformative change. Such initiatives are also used to foster a favourable image (ie, so-called health-washing or green-washing), narrow the focus of potential action, and deter state regulation.^57,61^ For example, sourcing sustainable palm oil, or reducing the amount of plastics used in ultra-processed food manufacturing is meaningless, if the products themselves are harmful to human health, superfluous to human need, and when markets for these products continue to grow.

From a performance standpoint, voluntary business initiatives are also questionable, given that competitive forces can deter sustained action. For example, in this Special Issue Trevena et al conduct a novel analysis of voluntary industry salt reduction actions. They reveal how the costs of doing business, competition and other forces linked with the external business environment makes these salt reduction initiatives largely ineffective.^62^ Robinson et al suggest that accountability initiatives that seek to monitor and improve food and beverage company policies and practices, can help to raise the visibility of nutrition and drive positive change within companies. However, in their analysis of such an initiative in Australia, they find company performance was limited, and that commitment to nutrition varied widely across companies, depending on their competitive positioning and willingness to change.^63^

###  Forms of Power

The third dimension of the Power Cube refers to forms of power, within and across levels and spaces. Here, we adopt instrumental, structural and discursive conceptualisations of power, given the comprehensive development and application of these concepts in the food and political science literatures.^18,19,52^ These are taken as heuristic devices, as these forms of power and their sources, clearly interact and overlap.

*Instrumental power* refers to the direct influence of one actor over another to affect decision-making and outcomes.^18,19^ This may include, for example, having access to decision-makers (eg, through lobbying activities, or shared participation in social networks), and intrinsic capacities (eg, leadership, strategic ‘soft power’ skills, technical capacities) that can be used to influence others. Much of the food politics literature refers to the instrumental power of ‘Big Food’ in undermining political commitment for action, and shaping food and nutrition policies through corporate lobbying activities. The growing (structural) market power of corporations amplifies and reinforces their instrumental power, as accumulating material resources can be used to fund political influence activities which, in-turn, create a favourable environment for ongoing consolidation.^26,38^ The ultra-processed food industry, for example, has strongly resisted policy responses using standard ‘playbook’ tactics.^64,65^ These include inter alia lobbying policy-makers, making political donations, adopting self-regulation to pre-empt and delay state action (policy substitution), public relations campaigns, and partnerships with community organizations.^52,64,66^ These activities often focus in ‘battleground’ jurisdictions, as shown in the intensive lobbying, media campaigns and corporate front groups resisting the adoption of sugar-sweetened beverage (‘soda’) taxes throughout Latin America.^67-69^ Moodie et al, in this Special Issue, demonstrate how industry groups have used these political practices to foster regulatory environments conducive to growing and sustaining ultra-processed food markets.^27^ Lacy-Nichols and Williams, further show how regulatory spaces are either being captured or contested for the purposes of cementing corporate interests, largely through ‘appeasement’ strategies, that frame corporations as ‘part of the solution,’ and through the co-option and partnership with health actors.^70^ Participation in MSPs further expands this corporate influence, by facilitating direct input into policy processes.^52,71^

*Structural power* is a more diffuse form of power, that manifests through the imposition of limits on the range of choices available to others, by cementing in instrumental and discursive capabilities and power resources, or creating institutional, ideational and policy environments in which certain ‘ways of doing things’ become normalized.^18,19^ It manifests through, for example, the establishment of formal processes and institutional structures (eg, laws, regulations), the shaping of informal norms and decision-making biases within policy-making institutions (ie, what beliefs and practices are considered acceptable and which are not). This form of power shapes the ‘decisions’ of actors but also their ‘non-decisions’ by implicitly selecting out alternative courses of action, and pre-defining preferences before any actual or visible decision-making process begins.^37,72^ Thow et al, in this issue, illustrate how the historical legacy of productivist and market-oriented food policies, have filtered out other priorities for sustainable food systems. Using Ghana as a case study, they show how a dominant food policy paradigm focused on economic growth, commodification and agricultural market expansion has been prioritised over nutrition and other social objectives in food policy-making processes.^73^ Similarly, in their analysis of Australian food and nutrition policy processes, Browne and colleagues find that a number of structural, institutional and ideational factors selectively filter the voices and knowledge systems of Indigenous people in or out of policy-making processes.^74^

Other papers in this series interrogate what regulatory approaches are required for transformative change. Parker et al provide an important critique of existing overly-circumscribed approaches, that alter minor system parameters in isolated ways through, for example, an emphasis on sustainable food labelling. They argue that transformative change, in contrast, will require an ‘ecological’ approach, involving packages of interventions that work synergistically throughout food systems, rather than piecemeal, sector or product specific ones.^75^ In their review of nutrition policies in high-income countries, Lee and colleagues findings support this critique, as policy responses have so far prioritised narrow voluntary industry initiatives on product reformulation and responsible marketing, partnerships with the commercial sector, and behaviour change communication targeting individuals. In contrast, upstream interventions targeting food supply chains and consumer food environments are absent, or notably weak.^76^ These preferences for voluntary and consumer-led interventions, at least to a significant extent, reflects the preferences and instrumental power of the food industry. Weak worldwide policy responses and the skew towards lifestyle-behavioural interventions also partly reflects the nature of food regulatory paradigms in many countries, and the structuring role of atomised consumer and risk logics of neoliberalism. For example, ‘cutting red-tape’ agendas and ‘nudge’ approaches to regulation have emerged in many high-income countries, with the potential to impede new food regulations targeting ultra-processed foods.^77^

An important example of structural power within food systems, is the increasing size and economic importance of food corporations in global and national economies.^19,26,31^ ‘Big Food,’ as providers of jobs, investments and tax revenues, can command substantial bargaining power with governments relative to small and medium-sized market actors. This can result in policy concessions that serve corporate interests, while enabling and reinforcing an industrial food systems paradigm.^26^ Economic globalization expands this power by making it easier to shift investments and production activities across borders, meaning governments must increasingly compete to attract and retain the investments and jobs TFCs provide.^19^ Market concentration, which has increased markedly at the global and national levels since the 1980s, further expands this form of power, whereby market share becomes increasingly consolidated in the hands of a declining number of firms operating within and across segments of the food supply chain, occurring largely through company mergers and acquisitions.^78^ Sievert et al, in their analysis of the politics of ‘meat reduction,’ find that just three corporations account for 63% of the global pork market, and two control 46% of the beef market.^79^

*Discursive power* is the power to shape underlying values, belief-systems (ie, world views and ideologies) and social norms, and the surface-level ‘frames’ and discourses through which food problems are interpreted and communicated.^18,19^ This is a more ‘hidden’ form of power that precedes and surrounds decision and non-decision making processes. It is the power to socialise others into accepting ‘truths’ about a given problem, and what problem interpretations and solutions are considered normal, acceptable and socially desirable. Actors can exercise discursive power through, for example, media engagement, public relations efforts and advertising. This power can also manifest in more structural forms, by shaping deeper sets of values, beliefs and norms within wider political systems, policy-making institutions and in society-at-large – for example the neoliberal belief in an expanded role for markets in governance, and the view that government should have no or only a minimal role in regulating free enterprise.

Commercial marketing is also an important form of discursive power within food systems, especially given the large marketing budgets of TFCs and the use of increasingly sophisticated digital marketing techniques.^27^ The development and extensive advertising of fortified, functionalised and reformulated foods is a direct response to rising concerns about the harms of ultra-processed foods, and reinforces a ‘nutrient-centric’ approach to nutrition that favours reformulation and other reductionist policy approaches.^80^ The appropriation of ‘alternative food movement’ discourses through, for example, organic, fair-trade or animal-welfare compliant product offerings, is an example of the adaptive capacity of corporations to exercise discursive power within food systems.^52,81^ The private standards and self-regulatory schemes within broader CSR programmes mentioned earlier generally portray corporations as ‘good corporate citizens’ and legitimise their role in food governance and their preferences for private standards over state regulation.^57,71^

 As a reflection of the outward shift in power described earlier, knowledge production in global food governance has also become increasingly devolved. The multi-lateral organizations of the UN System produce a diversity of knowledge products on a range of food systems issues, for example FAO’s State of Food and Nutrition Security in the World, UNICEF’s State of the World’s Children, and frequent reports of the HLPE-CFS. However, in recent decades there has been a significant increase in the number of non-state expert bodies and knowledge products, with different epistemological approaches and funding sources. These include, for example, the IPES-Food, the Food and Land Use Coalition, the Global Nutrition Report, and various Lancet commissions, among others. These have produced a diversity of knowledge products with varying, and in some cases conflicting, normative foundations and recommendations, suggesting that knowledge production itself has become increasingly politicised.

Indeed, defining sustainability and identifying what issues should be prioritised (or ignored) within food policy dialogue and action, and what trade-offs between objectives are needed or desirable, is deeply political. Some view sustainability as a ‘non-problem,’ as overly-complex or too costly to deal with; as the responsibility of individuals alone, or as a lesser priority relative to other objectives (eg, hunger reduction). A prominent ‘productivist’ view is that producing more food more efficiently to feed a rapidly growing population should be the primary (and potentially only) objective, with a significant role for agri-food technology. Some focus on shaping consumer demand through information provision (eg, labelling) or by using below-the-radar approaches focused on ‘choice-editing’ or ‘nudging.’ Others advocate for multi-issue and integrated approaches that mainstream sustainability into multiple food policy areas, including dietary guidelines.^42,82^ Contributions in this Special Issue largely support the view that sustainability problems are structurally determined, and arise because of asymmetrical relations of power between different actors and interests within food systems.^82^ Harris and Nisbett point to the importance of the basic determinants of malnutrition, and the need to challenge ideologies and belief systems that reinforce political processes of economic and social marginalisation, which in-turn, generate differential exposures and vulnerabilities to unhealthy food systems and malnutrition.^83^ Rose goes further, arguing that the core driver of today’s food systems crises is the logic of capitalism itself – ie, an unquestioned belief in and the strong institutionalisation of policies and practices that support continual growth and capital accumulation, profit maximisation, and processes of food commodification and financialisation, with market-based solutions to food problems sufficient in themselves.^84^

###  The Political Economy of Transformative Food Systems Change

A final theme in this Special Issue, is the political economy of food systems transformation. According to systems intervention frameworks, the extent to which ‘solutions’ generate transformational change depends not only on the type of intervention itself, but also where and at what level in the system it influences, and to what extent it works synergistically with other interventions.^85,86^ Often, food systems actors propose singular or a few interventions that tweak minor ‘system elements’ (eg, agricultural subsidies, sugar-sweetened beverage taxes, and food standards), which systems frameworks recognise as the least transformative forms of intervention, although when packaged together can generate significant effects. This contrasts with fully integrated or ‘ecological’ approaches to intervention,^75^ that modify multiple system structures (eg, national food policy councils, multi-level food policy infrastructures), that directly acknowledge and seek to redress power asymmetries (eg, through democratising food policy processes, or anti-trust policies targeting corporate concentration), or even challenge the deeper goals or ‘mental models’ of the system itself (eg, prioritising ecology, equity and human rights over the logic of neoliberal capitalism).

Different approaches to driving transformative change are acknowledged in this Special Issue. A first approach is to improve existing governance structures and institutions. Fanzo and colleagues, for example, suggest that public-private partnerships can work better for nutrition through more inclusive civil society participation, a focus on issues with clearer pathways to impact, and stronger accountability mechanisms.^56^ Robinson, Blake and Sacks suggest that accountability initiatives can help to monitor and benchmark the performance of corporate policies and practices in relation to health and nutrition, and help to make the case for stronger regulatory approaches involving government.^63^ Overall, evidence to support the effectiveness of corporate accountability initiatives and public-private partnerships appears to be limited. From a political economy and systems standpoint, there is a risk that such approaches fail to address, and may even reinforce existing power relations, by legitimising multi-stakeholder governance and private regulation, as a substitute for state intervention.

A second approach, puts forward counter-strategies to challenge the concentrated power of market actors. As noted by Lukes, a crucial reason to research power is to find ‘weak links’ though which to challenge power, a sentiment that resonates with the normative thrust of critical theory in political economy.^87^ Several contributions emphasise opportunities to challenge and overcome concentrated power in food systems. Friel, for example, building on a food systems power analysis, proposes strategies for civil society organizations and governments in the Global South, to counter concentrated and transnational corporate interests. These ‘weapons of the weak’ include coalition building, formulating ambitious and shared visions, strategically using multi-level institutional processes, civil society mobilization and organized campaigns, entrepreneurship, and compelling issue framing.^88^ Similarly, Moodie et al, drawing lessons from tobacco control, put forward suggestions for building powerful coalitions to counter the political activities of the ultra-processed food industry, including a much stronger role for state intervention, and diversifying the core public health training and skill set to include digital and political strategists, advocates, investigative journalists and lawyers.^27^ In order to safeguard regulatory space for governments within the global trade regime, Russ et al call for substantially expanding the participation of government health agencies and civil society organizations in the CAC.^89^

A third approach, calls for radical change, involving new social, economic and political food systems structures and relations of power. Rose puts forward how practices of food systems decommodification and commoning, under the organizing political principle of ‘food as a commons,’ are expanding in many localities worldwide, offering a counter-vailing force to the capitalist-industrial food system model and the crises that it is generating. This arguably seeks to transform the mental models and goals at the core of the food system itself, moving away from the capitalist logic of accumulation, commodification and perpetual growth.^84^ Harris and Nisbett call into question purely technical approaches to nutrition policy and programming action, often framed in terms of economics, scaling-up and investment. Such approaches largely fail to address the basic determinants of malnutrition, including processes of exclusion and marginalization of disadvantaged groups affected by malnutrition. They suggest two counter-vailing approaches for guiding thinking and action. First, a human rights-based approach involving political action towards greater entitlements and accountability, following the core principles of participation, accountability and non-discrimination in governance and service-delivery. Second, and similar to Rose, that realising the right to food can be further advanced through a commoning approach, involving collective forms of food production, consumption and governance, alongside burgeoning social movements grounded in food sovereignty and agroecology.^83^

## Conclusion

A number of important changes in food governance and power relations have occurred in recent decades with important implications for the sustainable food systems agenda. Although nation states retain their role as (arguably) the most important players in global and national food governance, there has been a ‘displacement’ of food system power in four main directions.^90^ First, *upwards* as globalization has given rise to more complex and globally integrated food systems governed increasingly by TFCs and international financial actors. Second, *downwards* as urbanization (including the rise of ‘mega-cities’) and decentralization of authority in many countries gives cities and sub-national actors a more prominent role in food governance. Third, *outwards* with a greater role for market and civil society actors facilitated by an expansion of food industry power and increasing preferences for market-orientated and multi-stakeholder forms of governance. Finally, power has also shifted *inwards* as markets have become increasingly concentrated through corporate strategies to gain and retain market power within and across food supply chain segments, within countries and globally. With this has come the massive expansion in the size and global reach of TFCs, and the power they wield in relation to both state and non-state actors. The expansion in the geographical scale and complexity of food systems, in the power of food corporations relative to nation states, and in the international rules governing national food economies has diminished the power of governments to influence food system activities both within and beyond their territorial borders. These changes, combined with the push for more devolved forms of multistakeholder governance, present significant risks as powerful transnational corporations, which lack democratic accountability, become increasingly dominant.

 The transformation of food systems ultimately requires an equally transformative, and even radical, change in the political economy of those systems. Technical ‘problem-solving’ and overly-circumscribed policy approaches that depoliticise food systems challenges, are insufficient to generate the change we need within the narrow time-frame we have. While there will be many paths to transformation, rights-based and commoning approaches hold great promise, based on principles of participation, accountability and non-discrimination, alongside social mobilization grounded in food sovereignty and agroecology. Finally, food systems are embedded within the broader and deeper structures of capitalism and, as several contributions to this collection argue, its recent neoliberal form. As some of the world’s countries slowly emerge from the shadow of the COVID-19 pandemic there have been multiple calls for a transformative shift in how we ‘build back fairer’ and not just ‘better.’ Indeed, there is reason to ditch the idea of ‘building back’ altogether since the economy we had before the pandemic was creating massive inequality and threatening imminent ecosystem collapse. The economy should not be ‘built back’ in any form, but transformed. Public health advocates for fundamental food systems change are not alone. There is a growing number of progressive social movements spanning social justice, environmental sustainability, gender empowerment, health equity, labour rights, and Indigenous sovereignty (to name but a few) that all, in differing ways, challenge the current form of capitalism as a system that is no longer ‘fit for purpose.’ The struggle for a new political economy for food systems thus aligns with many others, all seeking transformation into an eco-just political economy driven by the goals of ensuring a planet fit for human habitation, and participatory forms of governance fit for health and social equity.

## Ethical issues

 Not applicable.

## Competing interests

 Authors declare that they have no competing interests.

## Authors’ contributions

 PB, JLN, OW and RL conceptualised the Special Issue and this manuscript. PB and JL organized and facilitated the workshop informing this Special Issue. PB wrote the first draft of the manuscript and JLN, OW and RL provided critical input and review. PB, JLN, OW and RL managed the editorial review process for the 19 articles included in this Special Issue, with the exception of articles they authored. PB and RL provided financial contributions to support article processing and editing.

## Authors’ affiliations

^1^Institute for Physical Activity & Nutrition, Deakin University, Geelong, VIC, Australia.^2^Melbourne School of Population and Global Health, University of Melbourne, Melbourne, VIC, Australia. ^3^School of Politics and International Studies, University of Leeds, Leeds, UK. ^4^School of Public Health and Epidemiology, University of Ottawa, Ottawa, ON, Canada.

**Figure:**